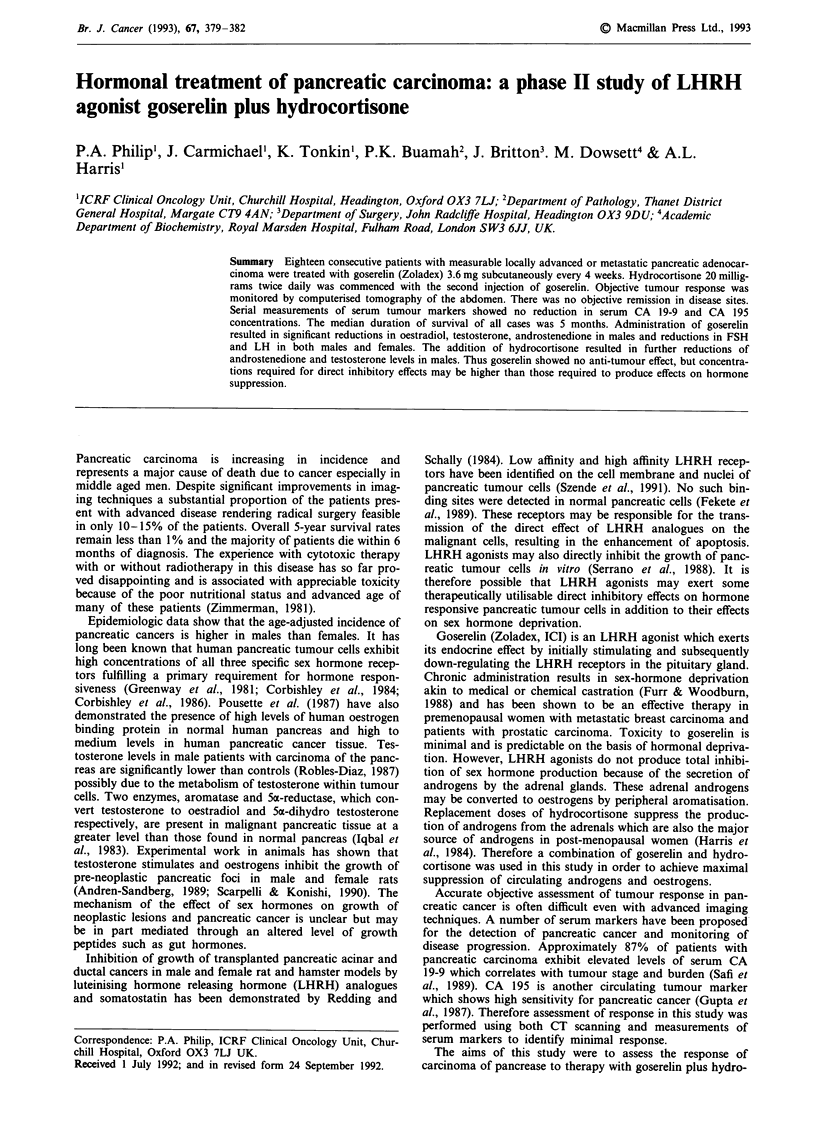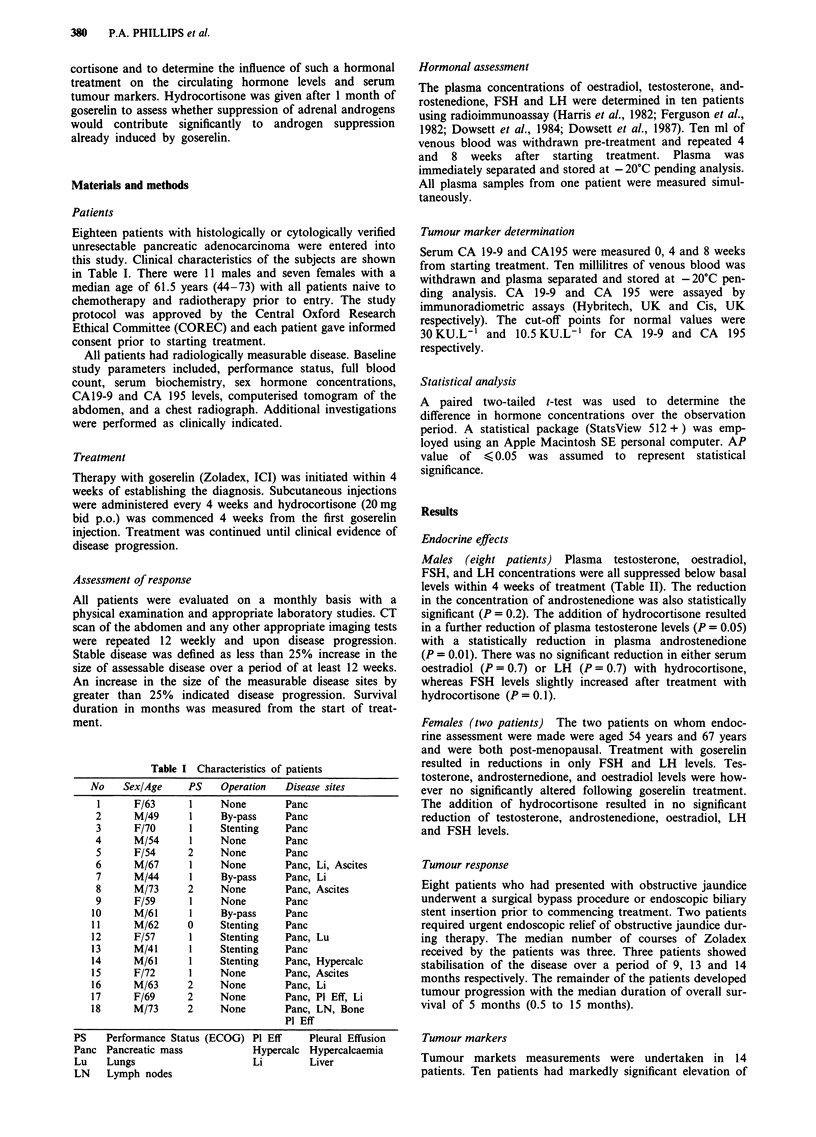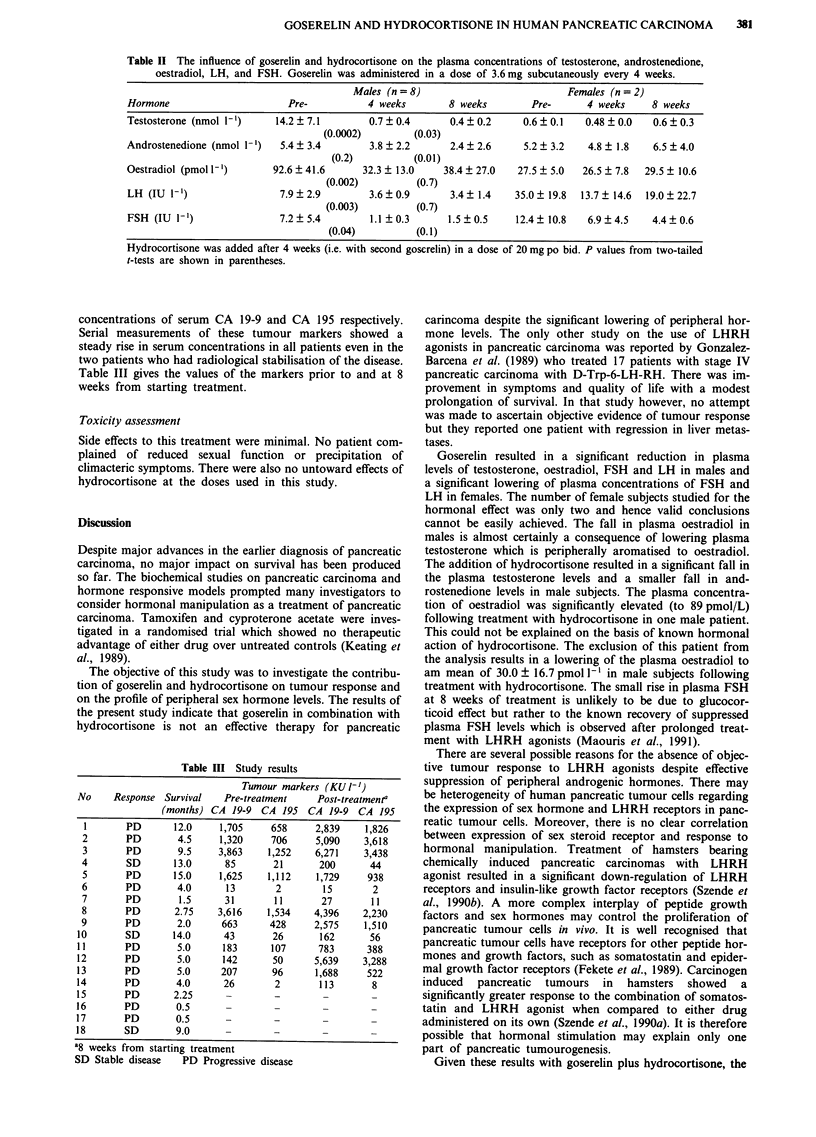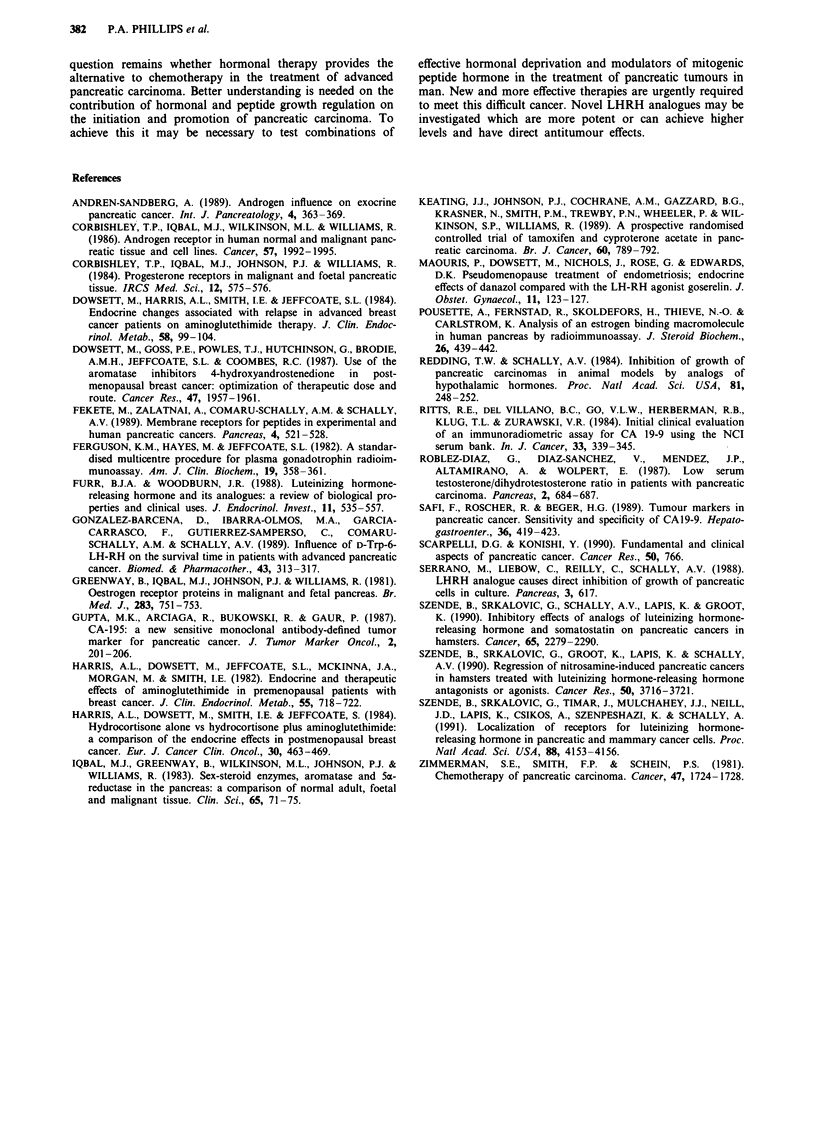# Hormonal treatment of pancreatic carcinoma: a phase II study of LHRH agonist goserelin plus hydrocortisone.

**DOI:** 10.1038/bjc.1993.69

**Published:** 1993-02

**Authors:** P. A. Philip, J. Carmichael, K. Tonkin, P. K. Buamah, J. Britton, M. Dowsett, A. L. Harris

**Affiliations:** ICRF Clinical Oncology Unit, Churchill Hospital, Headington, Oxford, UK.

## Abstract

Eighteen consecutive patients with measurable locally advanced or metastatic pancreatic adenocarcinoma were treated with goserelin (Zoladex) 3.6 mg subcutaneously every 4 weeks. Hydrocortisone 20 milligrams twice daily was commenced with the second injection of goserelin. Objective tumour response was monitored by computerised tomography of the abdomen. There was no objective remission in disease sites. Serial measurements of serum tumour markers showed no reduction in serum CA 19-9 and CA 195 concentrations. The median duration of survival of all cases was 5 months. Administration of goserelin resulted in significant reductions in oestradiol, testosterone, androstenedione in males and reductions in FSH and LH in both males and females. The addition of hydrocortisone resulted in further reductions of androstenedione and testosterone levels in males. Thus goserelin showed no anti-tumour effect, but concentrations required for direct inhibitory effects may be higher than those required to produce effects on hormone suppression.


Br. J. Cancer (1993), 67, 379-382 © Macmillan Press Ltd., 1993

### Hormonal treatment of pancreatic carcinoma: a phase II study of LHRH agonist goserelin plus hydrocortisone

P.A. Philip[1], J. Carmichael[1], K. Tonkin[1], P.K. Buamah[2], J. Britton[3]. M. Dowsett[4] & A.L. Harris[1]

[1]*ICRF Clinical Oncology Unit, Churchill Hospital, Headington, Oxford OX3 7LJ;* [2]*Department of Pathology, Thanet District General Hospital, Margate CT9 4AN;* [3]*Department of Surgery, John Radcliffe Hospital, Headington OX3 9DU;* [4]*Academic Department of Biochemistry, Royal Marsden Hospital, Fulham Road, London SW3 6JJ, UK.*

**Summary** Eighteen consecutive patients with measurable locally advanced or metastatic pancreatic adenocarcinoma were treated with goserelin (Zoladex) 3.6 mg subcutaneously every 4 weeks. Hydrocortisone 20 milligrams twice daily was commenced with the second injection of goserelin. Objective tumour response was monitored by computerised tomography of the abdomen. There was no objective remission in disease sites. Serial measurements of serum tumour markers showed no reduction in serum CA 19-9 and CA 195 concentrations. The median duration of survival of all cases was 5 months. Administration of goserelin resulted in significant reductions in oestradiol, testosterone, androstenedione in males and reductions in FSH and LH in both males and females. The addition of hydrocortisone resulted in further reductions of androstenedione and testosterone levels in males. Thus goserelin showed no anti-tumour effect, but concentrations required for direct inhibitory effects may be higher than those required to produce effects on hormone suppression.

Pancreatic carcinoma is increasing in incidence and represents a major cause of death due to cancer especially in middle aged men. Despite significant improvements in imaging techniques a substantial proportion of the patients present with advanced disease rendering radical surgery feasible in only 10–15% of the patients. Overall 5-year survival rates remain less than 1% and the majority of patients die within 6 months of diagnosis. The experience with cytotoxic therapy with or without radiotherapy in this disease has so far proved disappointing and is associated with appreciable toxicity because of the poor nutritional status and advanced age of many of these patients (Zimmerman, 1981).

Epidemiologic data show that the age-adjusted incidence of pancreatic cancers is higher in males than females. It has long been known that human pancreatic tumour cells exhibit high concentrations of all three specific sex hormone receptors fulfilling a primary requirement for hormone responsiveness (Greenway *et al.*, 1981; Corbishley *et al.*, 1984; Corbishley *et al.*, 1986). Pousette *et al.* (1987) have also demonstrated the presence of high levels of human oestrogen binding protein in normal human pancreas and high to medium levels in human pancreatic cancer tissue. Testosterone levels in male patients with carcinoma of the pancreas are significantly lower than controls (Robles-Diaz, 1987) possibly due to the metabolism of testosterone within tumour cells. Two enzymes, aromatase and 5α-reductase, which convert testosterone to oestradiol and 5α-dihydro testosterone respectively, are present in malignant pancreatic tissue at a greater level than those found in normal pancreas (Iqbal *et al.*, 1983). Experimental work in animals has shown that testosterone stimulates and oestrogens inhibit the growth of pre-neoplastic pancreatic foci in male and female rats (Andren-Sandberg, 1989; Scarpelli & Konishi, 1990). The mechanism of the effect of sex hormones on growth of neoplastic lesions and pancreatic cancer is unclear but may be in part mediated through an altered level of growth peptides such as gut hormones.

Inhibition of growth of transplanted pancreatic acinar and ductal cancers in male and female rat and hamster models by luteinising hormone releasing hormone (LHRH) analogues and somatostatin has been demonstrated by Redding and Schally (1984). Low affinity and high affinity LHRH receptors have been identified on the cell membrane and nuclei of pancreatic tumour cells (Szende *et al.*, 1991). No such binding sites were detected in normal pancreatic cells (Fekete *et al.*, 1989). These receptors may be responsible for the transmission of the direct effect of LHRH analogues on the malignant cells, resulting in the enhancement of apoptosis. LHRH agonists may also directly inhibit the growth of pancreatic tumour cells *in vitro* (Serrano *et al.*, 1988). It is therefore possible that LHRH agonists may exert some therapeutically utilisable direct inhibitory effects on hormone responsive pancreatic tumour cells in addition to their effects on sex hormone deprivation.

Goserelin (Zoladex, ICI) is an LHRH agonist which exerts its endocrine effect by initially stimulating and subsequently down-regulating the LHRH receptors in the pituitary gland. Chronic administration results in sex-hormone deprivation akin to medical or chemical castration (Furr & Woodburn, 1988) and has been shown to be an effective therapy in premenopausal women with metastatic breast carcinoma and patients with prostatic carcinoma. Toxicity to goserelin is minimal and is predictable on the basis of hormonal deprivation. However, LHRH agonists do not produce total inhibition of sex hormone production because of the secretion of androgens by the adrenal glands. These adrenal androgens may be converted to oestrogens by peripheral aromatisation. Replacement doses of hydrocortisone suppress the production of androgens from the adrenals which are also the major source of androgens in post-menopausal women (Harris *et al.*, 1984). Therefore a combination of goserelin and hydrocortisone was used in this study in order to achieve maximal suppression of circulating androgens and oestrogens.

Accurate objective assessment of tumour response in pancreatic cancer is often difficult even with advanced imaging techniques. A number of serum markers have been proposed for the detection of pancreatic cancer and monitoring of disease progression. Approximately 87% of patients with pancreatic carcinoma exhibit elevated levels of serum CA 19-9 which correlates with tumour stage and burden (Safi *et al.*, 1989). CA 195 is another circulating tumour marker which shows high sensitivity for pancreatic cancer (Gupta *et al.*, 1987). Therefore assessment of response in this study was performed using both CT scanning and measurements of serum markers to identify minimal response.

The aims of this study were to assess the response of carcinoma of pancrease to therapy with goserelin plus hydro-

Correspondence: P.A. Philip, ICRF Clinical Oncology Unit, Churchill Hospital, Oxford OX3 7LJ UK.
Received 1 July 1992; and in revised form 24 September 1992.

cortisone and to determine the influence of such a hormonal treatment on the circulating hormone levels and serum tumour markers. Hydrocortisone was given after 1 month of goserelin to assess whether suppression of adrenal androgens would contribute significantly to androgen suppression already induced by goserelin.

### Materials and methods

### Patients

Eighteen patients with histologically or cytologically verified unresectable pancreatic adenocarcinoma were entered into this study. Clinical characteristics of the subjects are shown in Table I. There were 11 males and seven females with a median age of 61.5 years (44–73) with all patients naive to chemotherapy and radiotherapy prior to entry. The study protocol was approved by the Central Oxford Research Ethical Committee (COREC) and each patient gave informed consent prior to starting treatment.

All patients had radiologically measurable disease. Baseline study parameters included, performance status, full blood count, serum biochemistry, sex hormone concentrations, CA19-9 and CA 195 levels, computerised tomogram of the abdomen, and a chest radiograph. Additional investigations were performed as clinically indicated.

### Treatment

Therapy with goserelin (Zoladex, ICI) was initiated within 4 weeks of establishing the diagnosis. Subcutaneous injections were administered every 4 weeks and hydrocortisone (20 mg bid p.o.) was commenced 4 weeks from the first goserelin injection. Treatment was continued until clinical evidence of disease progression.

### Assessment of response

All patients were evaluated on a monthly basis with a physical examination and appropriate laboratory studies. CT scan of the abdomen and any other appropriate imaging tests were repeated 12 weekly and upon disease progression. Stable disease was defined as less than 25% increase in the size of assessable disease over a period of at least 12 weeks. An increase in the size of the measurable disease sites by greater than 25% indicated disease progression. Survival duration in months was measured from the start of treatment.

**Table I** Characteristics of patients

| *No* | *Sex/Age* | *PS* | *Operation* | *Disease sites* |
|---|---|---|---|---|
| 1 | F/63 | 1 | None | Panc |
| 2 | M/49 | 1 | By-pass | Panc |
| 3 | F/70 | 1 | Stenting | Panc |
| 4 | M/54 | 1 | None | Panc |
| 5 | F/54 | 2 | None | Panc |
| 6 | M/67 | 1 | None | Panc, Li, Ascites |
| 7 | M/44 | 1 | By-pass | Panc, Li |
| 8 | M/73 | 2 | None | Panc, Ascites |
| 9 | F/59 | 1 | None | Panc |
| 10 | M/61 | 1 | By-pass | Panc |
| 11 | M/62 | 0 | Stenting | Panc |
| 12 | F/57 | 1 | Stenting | Panc, Lu |
| 13 | M/41 | 1 | Stenting | Panc |
| 14 | M/61 | 1 | Stenting | Panc, Hypercalc |
| 15 | F/72 | 1 | None | Panc, Ascites |
| 16 | M/63 | 2 | None | Panc, Li |
| 17 | F/69 | 2 | None | Panc, Pl Eff, Li |
| 18 | M/73 | 2 | None | Panc, LN, Bone Pl Eff |

PS Performance Status (ECOG); Pl Eff Pleural Effusion

Panc Pancreatic mass; Hypercalc Hypercalcaemia

Lu Lungs; Li Liver

LN Lymph nodes

### Hormonal assessment

The plasma concentrations of oestradiol, testosterone, androstenedione, FSH and LH were determined in ten patients using radioimmunoassay (Harris *et al.*, 1982; Ferguson *et al.*, 1982; Dowsett *et al.*, 1984; Dowsett *et al.*, 1987). Ten ml of venous blood was withdrawn pre-treatment and repeated 4 and 8 weeks after starting treatment. Plasma was immediately separated and stored at −20°C pending analysis. All plasma samples from one patient were measured simultaneously.

### Tumour marker determination

Serum CA 19-9 and CA195 were measured 0, 4 and 8 weeks from starting treatment. Ten millilitres of venous blood was withdrawn and plasma separated and stored at −20°C pending analysis. CA 19-9 and CA 195 were assayed by immunoradiometric assays (Hybritech, UK and Cis, UK respectively). The cut-off points for normal values were 30 KU.L$^{-1}$ and 10.5 KU.L$^{-1}$ for CA 19-9 and CA 195 respectively.

### Statistical analysis

A paired two-tailed *t*-test was used to determine the difference in hormone concentrations over the observation period. A statistical package (StatsView 512+) was employed using an Apple Macintosh SE personal computer. A$P$ value of $\leqslant 0.05$ was assumed to represent statistical significance.

### Results

### Endocrine effects

*Males (eight patients)* Plasma testosterone, oestradiol, FSH, and LH concentrations were all suppressed below basal levels within 4 weeks of treatment (Table II). The reduction in the concentration of androstenedione was also statistically significant ($P = 0.2$). The addition of hydrocortisone resulted in a further reduction of plasma testosterone levels ($P = 0.05$) with a statistically reduction in plasma androstenedione ($P = 0.01$). There was no significant reduction in either serum oestradiol ($P = 0.7$) or LH ($P = 0.7$) with hydrocortisone, whereas FSH levels slightly increased after treatment with hydrocortisone ($P = 0.1$).

*Females (two patients)* The two patients on whom endocrine assessment were made were aged 54 years and 67 years and were both post-menopausal. Treatment with goserelin resulted in reductions in only FSH and LH levels. Testosterone, androsternedione, and oestradiol levels were however no significantly altered following goserelin treatment. The addition of hydrocortisone resulted in no significant reduction of testosterone, androstenedione, oestradiol, LH and FSH levels.

### Tumour response

Eight patients who had presented with obstructive jaundice underwent a surgical bypass procedure or endoscopic biliary stent insertion prior to commencing treatment. Two patients required urgent endoscopic relief of obstructive jaundice during therapy. The median number of courses of Zoladex received by the patients was three. Three patients showed stabilisation of the disease over a period of 9, 13 and 14 months respectively. The remainder of the patients developed tumour progression with the median duration of overall survival of 5 months (0.5 to 15 months).

### Tumour markers

Tumour markets measurements were undertaken in 14 patients. Ten patients had markedly significant elevation of

**Table II** The influence of goserelin and hydrocortisone on the plasma concentrations of testosterone, androstenedione, oestradiol, LH, and FSH. Goserelin was administered in a dose of 3.6 mg subcutaneously every 4 weeks.

| Hormone | Males (n = 8) Pre- | 4 weeks | 8 weeks | Females (n = 2) Pre- | 4 weeks | 8 weeks |
|---|---|---|---|---|---|---|
| Testosterone (nmol $l^{-1}$) | 14.2 ± 7.1 | 0.7 ± 0.4 | 0.4 ± 0.2 | 0.6 ± 0.1 | 0.48 ± 0.0 | 0.6 ± 0.3 |
| | (0.0002) | (0.03) | | | | |
| Androstenedione (nmol $l^{-1}$) | 5.4 ± 3.4 | 3.8 ± 2.2 | 2.4 ± 2.6 | 5.2 ± 3.2 | 4.8 ± 1.8 | 6.5 ± 4.0 |
| | (0.2) | (0.01) | | | | |
| Oestradiol (pmol $l^{-1}$) | 92.6 ± 41.6 | 32.3 ± 13.0 | 38.4 ± 27.0 | 27.5 ± 5.0 | 26.5 ± 7.8 | 29.5 ± 10.6 |
| | (0.002) | (0.7) | | | | |
| LH (IU $l^{-1}$) | 7.9 ± 2.9 | 3.6 ± 0.9 | 3.4 ± 1.4 | 35.0 ± 19.8 | 13.7 ± 14.6 | 19.0 ± 22.7 |
| | (0.003) | (0.7) | | | | |
| FSH (IU $l^{-1}$) | 7.2 ± 5.4 | 1.1 ± 0.3 | 1.5 ± 0.5 | 12.4 ± 10.8 | 6.9 ± 4.5 | 4.4 ± 0.6 |
| | (0.04) | (0.1) | | | | |

Hydrocortisone was added after 4 weeks (i.e. with second goserelin) in a dose of 20 mg po bid. *P* values from two-tailed *t*-tests are shown in parentheses.

concentrations of serum CA 19-9 and CA 195 respectively. Serial measurements of these tumour markers showed a steady rise in serum concentrations in all patients even in the two patients who had radiological stabilisation of the disease. Table III gives the values of the markers prior to and at 8 weeks from starting treatment.

*Toxicity assessment*

Side effects to this treatment were minimal. No patient complained of reduced sexual function or precipitation of climacteric symptoms. There were also no untoward effects of hydrocortisone at the doses used in this study.

### Discussion

Despite major advances in the earlier diagnosis of pancreatic carcinoma, no major impact on survival has been produced so far. The biochemical studies on pancreatic carcinoma and hormone responsive models prompted many investigators to consider hormonal manipulation as a treatment of pancreatic carcinoma. Tamoxifen and cyproterone acetate were investigated in a randomised trial which showed no therapeutic advantage of either drug over untreated controls (Keating *et al.*, 1989).

The objective of this study was to investigate the contribution of goserelin and hydrocortisone on tumour response and on the profile of peripheral sex hormone levels. The results of the present study indicate that goserelin in combination with hydrocortisone is not an effective therapy for pancreatic carincoma despite the significant lowering of peripheral hormone levels. The only other study on the use of LHRH agonists in pancreatic carcinoma was reported by Gonzalez-Barcena *et al.* (1989) who treated 17 patients with stage IV pancreatic carcinoma with D-Trp-6-LH-RH. There was improvement in symptoms and quality of life with a modest prolongation of survival. In that study however, no attempt was made to ascertain objective evidence of tumour response but they reported one patient with regression in liver metastases.

Goserelin resulted in a significant reduction in plasma levels of testosterone, oestradiol, FSH and LH in males and a significant lowering of plasma concentrations of FSH and LH in females. The number of female subjects studied for the hormonal effect was only two and hence valid conclusions cannot be easily achieved. The fall in plasma oestradiol in males is almost certainly a consequence of lowering plasma testosterone which is peripherally aromatised to oestradiol. The addition of hydrocortisone resulted in a significant fall in the plasma testosterone levels and a smaller fall in androstenedione levels in male subjects. The plasma concentration of oestradiol was significantly elevated (to 89 pmol/L) following treatment with hydrocortisone in one male patient. This could not be explained on the basis of known hormonal action of hydrocortisone. The exclusion of this patient from the analysis results in a lowering of the plasma oestradiol to am mean of 30.0 ± 16.7 pmol $l^{-1}$ in male subjects following treatment with hydrocortisone. The small rise in plasma FSH at 8 weeks of treatment is unlikely to be due to glucocorticoid effect but rather to the known recovery of suppressed plasma FSH levels which is observed after prolonged treatment with LHRH agonists (Maouris *et al.*, 1991).

There are several possible reasons for the absence of objective tumour response to LHRH agonists despite effective suppression of peripheral androgenic hormones. There may be heterogeneity of human pancreatic tumour cells regarding the expression of sex hormone and LHRH receptors in pancreatic tumour cells. Moreover, there is no clear correlation between expression of sex steroid receptor and response to hormonal manipulation. Treatment of hamsters bearing chemically induced pancreatic carcinomas with LHRH agonist resulted in a significant down-regulation of LHRH receptors and insulin-like growth factor receptors (Szende *et al.*, 1990*b*). A more complex interplay of peptide growth factors and sex hormones may control the proliferation of pancreatic tumour cells *in vivo*. It is well recognised that pancreatic tumour cells have receptors for other peptide hormones and growth factors, such as somatostatin and epidermal growth factor receptors (Fekete *et al.*, 1989). Carcinogen induced pancreatic tumours in hamsters showed a significantly greater response to the combination of somatostatin and LHRH agonist when compared to either drug administered on its own (Szende *et al.*, 1990*a*). It is therefore possible that hormonal stimulation may explain only one part of pancreatic tumourogenesis.

Given these results with goserelin plus hydrocortisone, the

**Table III** Study results

| No | Response | Survival (months) | Tumour markers ($KU\ l^{-1}$) Pre-treatment CA 19-9 | CA 195 | Post-treatment[a] CA 19-9 | CA 195 |
|---|---|---|---|---|---|---|
| 1 | PD | 12.0 | 1,705 | 658 | 2,839 | 1,826 |
| 2 | PD | 4.5 | 1,320 | 706 | 5,090 | 3,618 |
| 3 | PD | 9.5 | 3,863 | 1,252 | 6,271 | 3,438 |
| 4 | SD | 13.0 | 85 | 21 | 200 | 44 |
| 5 | PD | 15.0 | 1,625 | 1,112 | 1,729 | 938 |
| 6 | PD | 4.0 | 13 | 2 | 15 | 2 |
| 7 | PD | 1.5 | 31 | 11 | 27 | 11 |
| 8 | PD | 2.75 | 3,616 | 1,534 | 4,396 | 2,230 |
| 9 | PD | 2.0 | 663 | 428 | 2,575 | 1,510 |
| 10 | SD | 14.0 | 43 | 26 | 162 | 56 |
| 11 | PD | 5.0 | 183 | 107 | 783 | 388 |
| 12 | PD | 5.0 | 142 | 50 | 5,639 | 3,288 |
| 13 | PD | 5.0 | 207 | 96 | 1,688 | 522 |
| 14 | PD | 4.0 | 26 | 2 | 113 | 8 |
| 15 | PD | 2.25 | – | – | – | – |
| 16 | PD | 0.5 | – | – | – | – |
| 17 | PD | 0.5 | – | – | – | – |
| 18 | SD | 9.0 | – | – | – | – |

[a]8 weeks from starting treatment
SD Stable disease PD Progressive disease question remains whether hormonal therapy provides the alternative to chemotherapy in the treatment of advanced pancreatic carcinoma. Better understanding is needed on the contribution of hormonal and peptide growth regulation on the initiation and promotion of pancreatic carcinoma. To achieve this it may be necessary to test combinations of effective hormonal deprivation and modulators of mitogenic peptide hormone in the treatment of pancreatic tumours in man. New and more effective therapies are urgently required to meet this difficult cancer. Novel LHRH analogues may be investigated which are more potent or can achieve higher levels and have direct antitumour effects.

### References

ANDREN-SANDBERG, A. (1989). Androgen influence on exocrine pancreatic cancer. *Int. J. Pancreatology*, **4**, 363-369.

CORBISHLEY, T.P., IQBAL, M.J., WILKINSON, M.L. & WILLIAMS, R. (1986). Androgen receptor in human normal and malignant pancreatic tissue and cell lines. *Cancer*, **57**, 1992-1995.

CORBISHLEY, T.P., IQBAL, M.J., JOHNSON, P.J. & WILLIAMS, R. (1984). Progesterone receptors in malignant and foetal pancreatic tissue. *IRCS Med. Sci.*, **12**, 575-576.

DOWSETT, M., HARRIS, A.L., SMITH, I.E. & JEFFCOATE, S.L. (1984). Endocrine changes associated with relapse in advanced breast cancer patients on aminoglutethimide therapy. *J. Clin. Endocrinol. Metab.*, **58**, 99-104.

DOWSETT, M., GOSS, P.E., POWLES, T.J., HUTCHINSON, G., BRODIE, A.M.H., JEFFCOATE, S.L. & COOMBES, R.C. (1987). Use of the aromatase inhibitors 4-hydroxyandrostenedione in post-menopausal breast cancer: optimization of therapeutic dose and route. *Cancer Res.*, **47**, 1957-1961.

FEKETE, M., ZALATNAI, A., COMARU-SCHALLY, A.M. & SCHALLY, A.V. (1989). Membrane receptors for peptides in experimental and human pancreatic cancers. *Pancreas*, **4**, 521-528.

FERGUSON, K.M., HAYES, M. & JEFFCOATE, S.L. (1982). A standardised multicentre procedure for plasma gonadotrophin radioimmunoassay. *Am. J. Clin. Biochem.*, **19**, 358-361.

FURR, B.J.A. & WOODBURN, J.R. (1988). Luteinizing hormone-releasing hormone and its analogues: a review of biological properties and clinical uses. *J. Endocrinol. Invest.*, **11**, 535-557.

GONZALEZ-BARCENA, D., IBARRA-OLMOS, M.A., GARCIA-CARRASCO, F., GUTIERREZ-SAMPERSO, C., COMARU-SCHALLY, A.M. & SCHALLY, A.V. (1989). Influence of D-Trp-6-LH-RH on the survival time in patients with advanced pancreatic cancer. *Biomed. & Pharmacother.*, **43**, 313-317.

GREENWAY, B., IQBAL, M.J., JOHNSON, P.J. & WILLIAMS, R. (1981). Oestrogen receptor proteins in malignant and fetal pancreas. *Br. Med. J.*, **283**, 751-753.

GUPTA, M.K., ARCIAGA, R., BUKOWSKI, R. & GAUR, P. (1987). CA-195: a new sensitive monoclonal antibody-defined tumor marker for pancreatic cancer. *J. Tumor Marker Oncol.*, **2**, 201-206.

HARRIS, A.L., DOWSETT, M., JEFFCOATE, S.L., MCKINNA, J.A., MORGAN, M. & SMITH, I.E. (1982). Endocrine and therapeutic effects of aminoglutethimide in premenopausal patients with breast cancer. *J. Clin. Endocrinol. Metab.*, **55**, 718-722.

HARRIS, A.L., DOWSETT, M., SMITH, I.E. & JEFFCOATE, S. (1984). Hydrocortisone alone vs hydrocortisone plus aminoglutethimide: a comparison of the endocrine effects in postmenopausal breast cancer. *Eur. J. Cancer Clin. Oncol.*, **30**, 463-469.

IQBAL, M.J., GREENWAY, B., WILKINSON, M.L., JOHNSON, P.J. & WILLIAMS, R. (1983). Sex-steroid enzymes, aromatase and 5α-reductase in the pancreas: a comparison of normal adult, foetal and malignant tissue. *Clin. Sci.*, **65**, 71-75.

KEATING, J.J., JOHNSON, P.J., COCHRANE, A.M., GAZZARD, B.G., KRASNER, N., SMITH, P.M., TREWBY, P.N., WHEELER, P. & WILKINSON, S.P., WILLIAMS, R. (1989). A prospective randomised controlled trial of tamoxifen and cyproterone acetate in pancreatic carcinoma. *Br. J. Cancer*, **60**, 789-792.

MAOURIS, P., DOWSETT, M., NICHOLS, J., ROSE, G. & EDWARDS, D.K. Pseudomenopause treatment of endometriosis; endocrine effects of danazol compared with the LH-RH agonist goserelin. *J. Obstet. Gynaecol.*, **11**, 123-127.

POUSETTE, A., FERNSTAD, R., SKOLDEFORS, H., THIEVE, N.-O. & CARLSTROM, K. Analysis of an estrogen binding macromolecule in human pancreas by radioimmunoassay. *J. Steroid Biochem.*, **26**, 439-442.

REDDING, T.W. & SCHALLY, A.V. (1984). Inhibition of growth of pancreatic carcinomas in animal models by analogs of hypothalamic hormones. *Proc. Natl Acad. Sci. USA*, **81**, 248-252.

RITTS, R.E., DEL VILLANO, B.C., GO, V.L.W., HERBERMAN, R.B., KLUG, T.L. & ZURAWSKI, V.R. (1984). Initial clinical evaluation of an immunoradiometric assay for CA 19-9 using the NCI serum bank. *In. J. Cancer*, **33**, 339-345.

ROBLEZ-DIAZ, G., DIAZ-SANCHEZ, V., MENDEZ, J.P., ALTAMIRANO, A. & WOLPERT, E. (1987). Low serum testosterone/dihydrotestosterone ratio in patients with pancreatic carcinoma. *Pancreas*, **2**, 684-687.

SAFI, F., ROSCHER, R. & BEGER, H.G. (1989). Tumour markers in pancreatic cancer. Sensitivity and specificity of CA19-9. *Hepatogastroenter.*, **36**, 419-423.

SCARPELLI, D.G. & KONISHI, Y. (1990). Fundamental and clinical aspects of pancreatic cancer. *Cancer Res.*, **50**, 766.

SERRANO, M., LIEBOW, C., REILLY, C., SCHALLY, A.V. (1988). LHRH analogue causes direct inhibition of growth of pancreatic cells in culture. *Pancreas*, **3**, 617.

SZENDE, B., SRKALOVIC, G., SCHALLY, A.V., LAPIS, K. & GROOT, K. (1990). Inhibitory effects of analogs of luteinizing hormone-releasing hormone and somatostatin on pancreatic cancers in hamsters. *Cancer*, **65**, 2279-2290.

SZENDE, B., SRKALOVIC, G., GROOT, K., LAPIS, K. & SCHALLY, A.V. (1990). Regression of nitrosamine-induced pancreatic cancers in hamsters treated with luteinizing hormone-releasing hormone antagonists or agonists. *Cancer Res.*, **50**, 3716-3721.

SZENDE, B., SRKALOVIC, G., TIMAR, J., MULCHAHEY, J.J., NEILL, J.D., LAPIS, K., CSIKOS, A., SZENPESHAZI, K. & SCHALLY, A. (1991). Localization of receptors for luteinizing hormone-releasing hormone in pancreatic and mammary cancer cells. *Proc. Natl Acad. Sci. USA*, **88**, 4153-4156.

ZIMMERMAN, S.E., SMITH, F.P. & SCHEIN, P.S. (1981). Chemotherapy of pancreatic carcinoma. *Cancer*, **47**, 1724-1728.